# The Enduring Gender Gap in STEM: A Meta-Analysis of Gender Differences in Self-Efficacy in STEM Fields

**DOI:** 10.3390/bs16010141

**Published:** 2026-01-19

**Authors:** Samantha L. McMichael, Stephen G. West, Virginia S. Y. Kwan

**Affiliations:** Department of Psychology, Arizona State University, Tempe, AZ 85281, USA

**Keywords:** STEM fields, gender gap, meta-analysis, self-efficacy

## Abstract

Women have made substantial gains in representation in some STEM fields (e.g., biology, chemistry, math) but not others (e.g., physics, computer science, engineering). Researchers have called for a STEM field-specific approach to investigate the persistent gender gap. While some studies indicate that males report higher self-efficacy than females, which may contribute to the persistent gender gap, other studies do not. The current research used Hunter–Schmidt meta-analysis to clarify the relationship between gender and self-efficacy in STEM fields where women are underrepresented compared to fields where representation has improved. A meta-analysis of 145 effects found gender differences in self-efficacy in all but one field (biology), but the magnitude of the difference was field-specific. In computer science and physics, two fields in which underrepresentation most strongly persists, there were greater gender differences in self-efficacy compared to the other fields. Findings also highlight participant educational stage as a potentially important factor in explaining heterogeneity of gender differences in self-efficacy within STEM fields and as an area for continued research.

## 1. Introduction

Growth of the workforce in science, technology, engineering, and mathematics (STEM) fields is widely recognized as a vital component of economic well-being in the United States (Fry et al., 2021; Martinez & Christnacht, 2021). Despite the necessity of workforce growth, there is a shortage of workers in these fields, a problem exacerbated by the continual underrepresentation of women in STEM. Extensive resources have been spent researching and designing interventions to address this societal issue, with large gains in some fields (e.g., biology and chemistry; Fry et al., 2021; Martinez & Christnacht, 2021; National Science Foundation, 2014, 2018), but not others (e.g., computer science, engineering, and physics; Fry et al., 2021; Martinez & Christnacht, 2021; National Science Foundation, 2014, 2018). For example, in the field of biology, women have continually earned the majority of bachelor’s degrees since 1988, whereas in computer science, women have not made significant representation gains since 1984 (National Science Foundation, 2014, 2018; Department of Education, 2021).

Research on the gender gap in STEM points to many potential explanatory factors. These include differences in cognitive ability, relative cognitive ability (i.e., relative strengths in math and verbal ability; Wang et al., 2013), lifestyle values, career interests, available role models, gender stereotypes, sexism/discrimination, and field-specific self-efficacy (see Ceci et al., 2009; Cheryan et al., 2017; Wang & Degol, 2017 for reviews). Although some of these explanatory factors suggest that the persistent gender gap in STEM is due to women’s choice (e.g., women choose other career fields because they are less interested in computer science than men), this narrow interpretation of the source of the gender gap does not address the underlying psychological factors that lead to women’s choices not to enter, or persist in, particular STEM fields (Cheryan et al., 2017; Gelernter, 1999; Henry, 2024). As research in this field develops and women have made gains in some, but not all, STEM fields, researchers have called for a STEM-field-specific approach to understand why women’s representation in some fields (e.g., biology, chemistry, mathematics) has improved whereas the large gender gap remains in others (e.g., computer science, engineering, physics; Cheryan et al., 2017).

Cheryan et al. (2017) provided a comprehensive narrative review of the literature on potential factors associated with the gender gap in STEM fields. They proposed a three-factor model—lack of early field-relevant experiences, reduced sense of belonging due to masculine culture, and gender gaps in field self-efficacy—that may explain differences in women’s representation across STEM fields (Cheryan et al., 2017). There is considerable evidence that self-efficacy—self-judgments about one’s skills and abilities to succeed—influences behavior by guiding activity choice, effort, persistence, and performance skill level (Bandura, 1977, 1981; Schunk, 1984). In academics, greater self-efficacy predicts grade striving, self-regulation, effective time management, less academic disengagement, higher GPAs, and college retention (Liem & Martin, 2012; Robbins et al., 2004; Zimmerman et al., 1992). A meta-analysis including 109 studies reported that academic self-efficacy was the strongest psychological predictor of both GPA and retention in higher education (Robbins et al., 2004). The benefits of self-efficacy in academics in general are well-documented, and self-efficacy is potentially experimentally malleable, making it a good target for intervention (Cervone & Peake, 1986).

Although Cheryan et al. (2017) included self-efficacy in their explanatory model for variation in the gender gap by STEM field, the evidence for self-efficacy was substantially weaker than the evidence supporting the other two factors. Cheryan et al. (2017) noted that the research evidence was mixed for the presence of larger gender differences in self-efficacy in STEM fields where women remain underrepresented. Specifically, some studies reported relatively lower self-efficacy for women as compared to men in STEM fields with larger gender gaps (e.g., Cheryan & Plaut, 2010; Matskewich & Cheryan, 2016; Rosson et al., 2011), whereas other studies did not (e.g., Concannon & Barrow, 2009; Lent et al., 2005). Overall, although many studies have explored gender differences in STEM self-efficacy, further synthesis is needed to assess the magnitude of gender differences in STEM fields where women remain underrepresented.

Past meta-analyses have explored gender differences in general academic self-efficacy and in some specific STEM fields. A meta-analysis of gender differences found a small difference favoring males in overall academic self-efficacy (Hedge’s *g* = 0.08; Huang, 2013). Female students also had higher self-efficacy in language arts than males, whereas male students had higher math and computer self-efficacy. No gender difference was found in general “science” self-efficacy. Early meta-analyses of computer and math self-efficacy found a weak to moderate effect suggesting that male students had higher computer and math self-efficacy than females (Whitely, 1997; Hyde et al., 1990). These analyses also provided support for differences by student age, finding that gender differences in computer self-efficacy were greater in high school students than elementary or university students and differences in math self-efficacy increased by age. In a more recent meta-analysis (i.e., articles published by 2016), Parker et al. (2020) found a small advantage for boys in math success expectancies and a smaller, but still significant, advantage in general science. This meta-analysis provided additional support for differences in gender effects by specific STEM field. For example, it found a larger difference in expectancies of success if the domain measured was specific to computer science and no difference in expectancies of success in biology-specific measures. Parker et al. (2020) provided strong initial support for STEM field-specific gender differences; however, the meta-analysis limited its coverage to articles that focused on both self-concept and task value and measured gender differences in both variables, limiting the number of findings included in the meta-analyses.

The present meta-analysis builds on the foundation of the prior narrative review by Cheryan et al. (2017) and prior meta-analyses to help illuminate the role of self-efficacy in the gender gap in STEM fields. We examined the same six STEM fields considered by Cheryan et al. (2017): three (computer science, engineering, physics) in which women are underrepresented, and three (biology, chemistry, mathematics) in which substantial gains in representation have occurred. In their narrative review, Cheryan et al. (2017) concluded that the results regarding the association of the STEM gender gap and self-efficacy were mixed. As originally illustrated by Hunter and Schmidt (1980); (see also Borenstein et al., 2021; Schmidt & Hunter, 2017; Valentine et al., 2019), meta-analysis can help clarify inconsistent differences in the research literature by eliminating study artifacts and supporting the examination of potential moderator effects. We provide a comprehensive meta-analytic review of Cheryan et al.’s hypothesis that gender differences in self-efficacy are greater in STEM fields where the gender gap persists. We investigate two potential moderators—domain of self-efficacy and participant educational stage—in an attempt to provide insight into both the optimal STEM fields and timing for potential intervention to reduce gender differences in STEM interest and persistence.

### 1.1. Domain of Self-Efficacy

The social-cognitive theoretical foundations of self-efficacy suggest that perceived competence is domain-specific, and research on self-efficacy varies in its focal domain (Bandura, 1977, 1981). Differences in focal domain may contribute to the mixed findings in STEM self-efficacy research (i.e., gender differences may vary by specific STEM field). An aim of the present meta-analysis was to determine if reported gender differences in STEM self-efficacy vary by STEM field (i.e., the self-efficacy domain). To provide clarity on the role of STEM fields, this research sought to minimize the variability of other aspects of domain specificity. Specifically, research on self-efficacy in different STEM fields varies widely in domain specificity in two areas: measurement of self-efficacy and participant background.

In STEM research, studies investigating self-efficacy employ various measures that differ in their domain specificity. For example, a study may focus on general academic self-efficacy, mathematics self-efficacy, or self-efficacy in a specific field. This difference in measurement domain may lead to confusion about the magnitude of gender differences in self-efficacy. For example, students in STEM may be generally high academic achievers (Park et al., 2007). In studies measuring academic self-efficacy, gender differences in self-efficacy ratings may be small and/or not statistically significant. In a study of students with high aptitude for math (i.e., students who may be well-suited for STEM), women were more likely to also have high verbal aptitude (Wang et al., 2013), suggesting that they may report high perceived competence in general academics. In contrast, in STEM fields where women are still underrepresented (e.g., physics), women may rate their self-efficacy in that specific field lower in comparison to men. Domain-specific differences could be due to many factors related to aspects of particular STEM fields (e.g., salience of gender stereotypes; lack of similar role models; masculine culture). Differences in which domain researchers measure may account for mixed findings in the STEM self-efficacy research and make it unclear if there are gender differences in self-efficacy by specific field.

#### Participant Background

Research on STEM self-efficacy also varies in the background and situational context of the participants. For example, some researchers may use a convenience sample of psychology undergraduates to measure computer science self-efficacy, whereas others may focus on students who are currently enrolled in a computer science course or are computer science majors. Sources of self-efficacy are largely dependent on experiences with that specific domain (Bandura, 1977, 1981). In fact, the four main sources of an individual’s self-efficacy—mastery experiences, vicarious experiences (e.g., observing role models), social persuasion, and emotional and physiological states—are founded on an individual’s experience within a domain. Additionally, some literature points to gender differences in the role of those four sources in developing self-efficacy (e.g., men relying more on personal mastery and women on social influences (Usher, 2009); see also Usher & Pajares, 2006; Zeldin et al., 2008; Zeldin & Pajares, 2000).

As a result, reported findings on gender differences in STEM self-efficacy may differ depending on the amount of experience participants have with a specific STEM field. For example, measuring physics self-efficacy in a student sample from a psychology course (i.e., students who may have little to no experience in physics) may provide different findings than a study of self-efficacy in students currently enrolled in a physics course. In contrast, gender differences may be more stable in studies measuring self-efficacy in a specific domain using a sample of students currently engaged in that domain (e.g., enrolled in a course or the major). These differences in participant backgrounds may lead to mixed findings in the research.

To test if gender differences in self-efficacy vary by STEM fields, the current meta-analysis minimized sources of variability in domain specificity. Specifically, we focused on studies that measured field-specific self-efficacy in participants currently involved in the STEM fields of interest. For example, potential studies for inclusion may measure physics self-efficacy in a physics course, chemistry self-efficacy for chemistry majors, or math self-efficacy in elementary students. Additionally, due to differences across cultures (e.g., norms of gender socialization; structure and norms of education systems), this meta-analysis focused on studies measuring self-efficacy in the United States. These approaches of minimizing variability allowed for a focused analysis of self-efficacy differences between males and females in three subgroups: (1) all STEM fields combined, (2) underrepresented fields versus representation-improved fields, and (3) each STEM field separately.

### 1.2. Educational Stage of Participants

Research on gender and self-efficacy in STEM includes participants ranging from elementary school to adults who are established in their careers. Although gender differences in STEM self-efficacy have been documented across educational levels (e.g., Hyde et al., 1990; Fredricks & Eccles, 2002; Sax, 1994), the magnitude of the differences may vary, possibly contributing to the perception of mixed findings. Potential changes in the magnitude of gender differences in STEM self-efficacy over time may be related to features of the education system (e.g., factors that facilitate continuous stereotype threat) and/or developmental changes over time (e.g., gender roles and societal expectations; Henry & Sears, 2009).

Driven by preconceptions, women in STEM are at risk of experiencing stereotype threat (i.e., the perception that they may perform poorly and confirm the negative stereotype that women as a group are not as capable in STEM fields), leading to hampered academic performance (Steele & Aronson, 1995). Endorsing traditional gender stereotypes of women’s math abilities predicts a woman’s own math self-efficacy and math task performance (Bonnot & Croizet, 2007). Although the negative impact of stereotype threat has been demonstrated in even young elementary students (Huguet & Régner, 2007; Muzzatti & Agnoli, 2007), some research indicates that the negative effects of continuous stereotype threat may accumulate as students continue in their education (Bedyńska et al., 2019). Additionally, as women advance in STEM fields, particularly in fields where women remain vastly underrepresented, they may feel more pressure to perform well as an example of their group and therefore may be more susceptible to stereotype threat (Steele & Aronson, 1995). As a result, there may be gradual increases in gender differences in self-efficacy, particularly as students move through their educational careers.

From a developmental perspective, gender differences in self-efficacy may significantly increase as students transition into university. As students enter college, they enter a life stage—“emerging adulthood”—that is defined as a time of identity exploration and formation (Arnett, 2000). During this stage, men and women encounter different social and biological expectations. Young women begin to face conflicting societal and biological demands to emphasize future family or career roles (Amatea et al., 1986). As women continue through this life stage they may anticipate upcoming life changes and conflicts. Their perception of their competence to succeed in STEM fields, particularly those without sufficient similar role models for success, may diminish. As such, as college-aged women begin to consider their future lives, the self-efficacy gender gap may increase. This difference may be particularly evident depending on which domain of self-efficacy researchers assess (e.g., computer science self-efficacy or biology self-efficacy).

Prior research and meta-analyses reporting on gender differences in STEM self-efficacy by educational level have reported seemingly mixed findings. As stated above, an early meta-analysis (Hyde et al., 1990) of differences in math self-confidence found larger differences by gender as students progressed in educational level (e.g., women were at a greater disadvantage in high school and university than in elementary school). In contrast, one study spanning from primary to secondary education found that the gender gap in mathematics self-efficacy lessened with increased years of education (Fredricks & Eccles, 2002). In computer science, Whitely (1997) reported that gender differences in self-efficacy were larger for high school students than for elementary and university students. Investigating age as a moderator for various domains of self-efficacy (e.g., math, general science, computer science, biology), another meta-analysis found age was only a significant moderator for the general science domain (Parker et al., 2020). Specifically, gender differences in general science self-efficacy in elementary school were not statistically significant, but the difference favoring men became significant by young adulthood. Parker et al. (2020) noted that there were few findings for some of the age ranges (e.g., elementary school age), which may account for the non-significant findings in the other domains.

Overall, these inconsistencies in the reported impact of educational stage as a moderator of the relationship between gender and STEM self-efficacy suggest that further analysis is necessary. While educational stage is theoretically relevant across STEM fields, the ability to test the potential moderation effect of educational stage within individual STEM fields relies on the available data in the literature. For example, if the current literature included few reported effects for computer science self-efficacy in elementary-aged students, a meta-analysis considering educational stage as a moderator would be underpowered. However, a finding that there are few results in the literature for particular educational stages would be instructive and highlight the need for future research in certain STEM fields. As such, the current research did not limit inclusion by age and collected all available reported findings for all ages to facilitate testing educational stage as a potential moderator, when possible, given the available data.

### 1.3. Date of Study

Gender differences in STEM self-efficacy may be changing over time. For example, potential changes in the magnitude of gender differences in STEM self-efficacy might have occurred as a function of changes in the gender composition of fields, the gender composition of faculty, or changes in attitudes and societal expectations over time (see Cheryan et al., 2017). This may be particularly true in fields where women have made gains in representation (e.g., biology, math, chemistry). When studying a phenomenon that may be changing, meta-analyses often consider the date of the study (e.g., Cooper, 2017; Shadish et al., 2000). To investigate the possibility of differences in effect sizes by when the study was conducted, we tested the date of the study as a predictor of differences in the magnitude of the STEM self-efficacy gender gap.

### 1.4. Aim of the Current Research

The current research aimed to clarify the relationship between gender and self-efficacy in STEM fields where women are currently underrepresented (i.e., computer science, engineering, physics) compared to fields where representation has improved (i.e., math, biology, chemistry). This research followed Cheryan et al. (2017) in grouping computer science, engineering, and physics as STEM fields where women are currently underrepresented and math, biology and chemistry as fields where women’s representation has improved. This grouping is based on both the current percentage of bachelor’s degrees awarded in the U.S. to women (i.e., at or near 50% for representation-improved) and the overall trend in representation over time. See Cheryan et al. (2017) for a more complete discussion. Specifically, the present meta-analysis aimed to integrate the existing findings in the literature to determine if there is evidence for gender differences in self-efficacy in specific STEM fields, if those differences vary by women’s representation in the fields, and the magnitude of those differences. This research used Hunter–Schmidt meta-analysis, which has a particular strength in differentiating whether differences in findings across studies are best explained by study artifacts (e.g., sampling error) or the presence of critical moderators (i.e., the domain of self-efficacy measured and/or educational level of participants).

To achieve this aim, this research used Hunter–Schmidt meta-analysis (Hunter & Schmidt, 1980; Schmidt & Hunter, 2017) to capitalize on the rich existing literature on gender and self-efficacy in STEM. Using meta-analysis allowed this research to (1) clarify the underlying relationships between gender and self-efficacy in STEM fields and (2) identify whether the source of the mixed findings in the literature was due to study artifacts alone or to the presence of moderators that may be critical to theory building and future intervention.

## 2. Materials and Methods

This research conducted a meta-analysis to synthesize the findings on the relationship between gender and self-efficacy in STEM fields where women are currently underrepresented (i.e., computer science, engineering, physics) compared to each of the fields where representation has improved (i.e., biology, chemistry, math). The search terms and strategies for collecting citations for potential inclusion are described below.

### 2.1. Search Strategy to Identify Articles

We followed general meta-analytic search strategies as outlined by Cooper (2017), White (2019), and Glanville (2019).

- Databases: PsycInfo, ERIC, Engineering Village, PubMed^1^
- Search Terms: (“Gender Difference*” OR “Sex Difference*”) AND (STEM OR Scien* NOT “STEM Cell”) AND (Biolog* OR Chemist* OR Math* OR “Computer Scien*” OR Computing OR Engineer* OR Physic* NOT Physician NOT Physical) AND (“self-efficacy” OR “self efficacy” OR “anticipated success” OR confiden* OR “expectancy for success” OR competenc* OR “academic self-concept” OR “academic self concept”)
- Additional Review Citations: In addition to the database searches, we also collected potential articles cited by recent review papers (Ceci et al., 2009; Cheryan et al., 2017; Wang & Degol, 2017). Finally, to collect more recent articles that may focus on gender differences in self-efficacy in specific STEM fields (rather than STEM in general), we collected articles in a descendancy search that cited Cheryan et al. (2017).

Citations were stored in EndNote (The EndNote Team, 2013, Version X9) and exact duplicates were removed.

### 2.2. Method of Reviewing Studies for Inclusion

**Figure 1:**
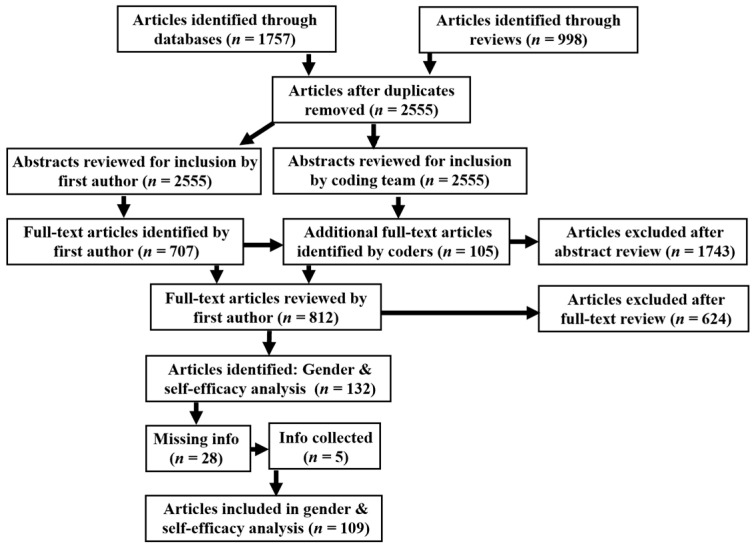
Flow Diagram for Meta-Analyses Article Identification. *Note*. *n* = number of articles.

Due to the large number of collected articles (*n* = 2555), our research team first reviewed abstracts for potential inclusion (see the flow diagram; Figure 1) based on the full inclusion criteria (Table 1). To reduce bias and error in identifying studies for inclusion, two coders—the first author and a member of the coding team— read each collected abstract and indicated if the full-text article should be reviewed based on the inclusion criteria (Cooper, 2017). Coding was performed by the first author and a seven-person coding team. The first author coded every abstract and each member of the coding team coded between 353 and 440 abstracts. To code each abstract, each coder first read the abstract and then answered six “yes or no” questions based on the study inclusion criteria (see the coding guide questions in Supplementary A in Online Resource S1^2^). Abstracts with “yes” answers on all six questions were identified for full-text review. The average reliability between the first author and each of the members of the coding team was high (Mean Cohen’s *Kappa* = 0.858; *Kappa* ranged from 0.786 to 0.923 between the first author and the seven coders; Cohen, 1960).

Study abstracts identified by one of, or both of, the first author and the coding team member were included in the full-text article review.^3^ The first author read the full texts of all articles short-listed by the coders (*n* = 812) according to the inclusion criteria and then collected the targeted effects, if reported in the article (see Supplementary B in Online Resource S1 for the coding guide). After reviewing all full-texts, 132 articles were identified that both met the inclusion criteria and reported effects for gender differences in STEM field self-efficacy. In some cases, articles reporting relevant effect sizes were missing crucial information for inclusion in the meta-analysis (*n* = 28). In these cases, the researchers attempted to collect the information from authors; the information was successfully collected in 5 out of 28 cases (18%). See Online Resource S1 for full information on methods of attempting to collect missing information.

### 2.3. Articles with Multiple Reported Effects

Some articles contained multiple measures, considered multiple STEM fields, and/or contained multiple studies relevant to the current meta-analysis. In these cases, articles may have contributed more than one effect to the meta-analysis. See Online Resource S1 for information on how those situations were handled in the analyses. See Table 2 for the number of included effects by number of articles.

### 2.4. Data Analysis Method

The studies identified by the inclusion criteria were collected using EndNote (The EndNote Team, 2013, Version X9). The research team collected study effect sizes (Cohen’s (1988) d and/or Pearson’s r as relevant), precision (i.e., reliability of measures), domain of self-efficacy measured, age of participants, date of study, and other relevant statistics for artifact correction (see the Corrections for Error and Bias section below). After correcting each effect for error and bias, the effects were aggregated to form the mean corrected effect and confidence interval for each meta-analysis using the Hunter–Schmidt Meta-Analysis Programs Package (V.2; Schmidt & Le, 2014)^4^. We used a random effects model, which assumes the true effect size varies across the studies in the meta-analysis. Random effects meta-analysis is the preferred method when researchers have used different methods to investigate the relationship of interest (Raudenbush, 2009; Schmidt & Hunter, 2017).

This research assessed variation among the study effect sizes using (a) the 80% credibility interval and (b) the percentage of variance in effects that was attributed to artifacts (i.e., sampling and measurement error). The credibility interval refers to the distribution of parameter values and provides a meaningful understanding of the variability of effect sizes. The percentage of variance attributed to artifacts provides an understanding of the proportion of the variability in effect sizes that may be due to meaningful variation in effects rather than artifacts alone. If the percentage of variance attributed to artifacts was below 75% (i.e., the heterogeneity in effect sizes not attributed to artifacts alone was greater than 25%), we concluded that there was evidence for heterogeneity of the study effect sizes (Schmidt & Hunter, 1977). If variation among effect sizes remained after accounting for artifacts, we used sub-group meta-analysis to test if the proposed moderators (i.e., self-efficacy domain, educational stage of participants, date of study^5^) led to different effect sizes^6^ and accounted for a portion of the remaining variation in effects.

The results of the meta-analysis were displayed graphically using forest plots, which display the study effect sizes sorted by sample size (largest to smallest), confidence intervals, and the average corrected effect size for the effects in the meta-analysis (Cooper, 2017). These plots allow for identification of potential outliers and publication bias (i.e., average effect sizes that become systematically larger as sample sizes decrease, indicating potential publication bias; Schmidt & Hunter, 2017). All plots are available in Online Resource S1.

### 2.5. Corrections for Error and Bias

The meta-analysis literature outlines eleven types of study artifacts that may be sources of error and bias in meta-analyses (Schmidt & Hunter, 2017). Each of these artifacts and their relevance to this research are detailed in Supplementary C in Online Resource S1 (see Schmidt & Hunter, 2017) for a more complete discussion of study artifacts and corrections). Three artifacts—sampling error and measurement error in both the independent and dependent variables—are present in all studies and should always be corrected for in meta-analyses.

The current meta-analyses corrected for these three artifacts according to the recommendations in Schmidt and Hunter (2017). To correct for measurement error, coefficient alpha for the independent and dependent variables was collected. The majority (77%) of the included studies reported coefficient alpha for the dependent variable and independent variable (when applicable). In cases in which coefficient alpha was not reported: (1) the value was identified through another article that used the same measure (42% of missing cases), or (2) the average coefficient alpha for all measures of the same construct was imputed.

The remaining artifacts may or may not be present in the sample of studies that make up a specific meta-analysis. No correction was performed for these artifacts in the current meta-analysis. See Online Resource S1 for a discussion of these artifacts. Consequently, the variances of effects reported in the current meta-analyses should be considered the maximum possible true variation between study effects (Schmidt & Hunter, 2017).

### 2.6. Relationship Between Study Date and Effect Size

For each of the six STEM fields, we constructed scatterplots of the relationship between the date of the study and the effect size. Inspection of the scatterplots did not indicate outliers. Inspection of the fit of lowess smooths (Cleveland, 1981) suggested a linear relationship was adequate to represent the relationship. We then calculated Pearson correlations between the date of the study and the effect size to assess whether the magnitude of the effect size changed over time.

### 2.7. Transparency and Openness

This research followed the APA Style Meta-Analytic Reporting Standards (MARS) (Appelbaum et al., 2018) and was preregistered at Open Science Framework (https://osf.io/xvfgb/?view_only=f4bd749c923b4f2994de41fe66d458ea). The data are available to download at https://osf.io/jt3a4/?view_only=3109a214794d406b8a3e2ef0837d8a43. Data were analyzed using Hunter–Schmidt Meta-Analysis Programs Package (V.2; Schmidt & Le, 2014).

## 3. Results

### 3.1. Descriptive Statistics for Included Effects

Table 2 reports the number of included effects by STEM field. Original articles with studies included in the meta-analysis are identified in the references. Publication dates ranged from 1991 to 2023. Approximately 88 percent of the included articles were published in 2000 or after, and approximately 73 percent were published in 2010 or after (see Supplementary D in Online Resource S1). The average proportion of women in the samples of the studies reviewed varied by STEM domain: biology = 0.61, chemistry = 0.60, math = 0.50, physics = 0.44, computer science = 0.37, and engineering = 0.36. Using the proportion of women earning bachelor’s degrees as a reference, women may have been oversampled in studies in the fields in which women are designated as still underrepresented (i.e., physics, computer science, engineering; see Supplementary D in Online Resource S1; National Science Foundation, 2018). The articles varied in whether and how they reported the racial breakdown of their samples. Approximately 67 percent (*n* = 97) provided a measure of the proportion of white participants. The average proportion of white participants was 0.66 (range: 0.00–0.98).

### 3.2. Gender Differences in Self-Efficacy in STEM

As presented above, prior literature reviews suggested that there are mixed findings in the literature for gender differences in STEM self-efficacy (i.e., some found differences favoring men, some found differences favoring women, and others found no gender difference). For an initial view, Table 3 reports the statistical significance of the gender differences in self-efficacy by STEM field for the effects in the current meta-analysis. Across the six STEM fields, few studies reported effects favoring women (approximately 2% of total effects), 32% reported no statistically significant gender effect, and 66% reported statistically significant effects favoring men. This finding, although descriptive in nature, supports further investigation of potential sources of variation across effects. Additionally, there was a statistically significant overall difference in reported findings across the six specific STEM fields, suggesting that there is variation in self-efficacy effects across fields, *χ*^2^(10, *N* = 144) = 20.96, *p* = 0.021.

These descriptive results are consistent with the mixed findings in the literature and differential gender differences by STEM field. Hunter–Schmidt meta-analysis can help us understand the magnitude and variation in the overall effect and determine if differences in significance are due to artifacts in the studies. Below, we first consider all six STEM fields combined to test the potential explanation that true gender differences in self-efficacy do not vary across fields and that any variation in effects is due to artifacts (i.e., sampling error, measurement error).

#### 3.2.1. Meta-Analysis

##### All STEM Fields Combined

Table 4 presents the full results for the meta-analysis of gender differences in self-efficacy across the six STEM fields combined. Overall, men had higher self-efficacy compared to women in the STEM fields. The effect was between small and medium in magnitude (Cohen, 1988), *d* = 0.308. The 80% credibility interval suggests a range in effects from virtually no effect (*d* = 0.050) to a medium effect size (*d* = 0.566). Only a small percentage (10.1%) of variation in the effects was due to artifacts. This suggests that moderators may account for variation in effects. Based on this finding, we tested for variation in gendered self-efficacy differences by women’s representation in STEM (i.e., are there differences between fields in which women are underrepresented and fields in which representation has improved?).

##### Underrepresented Versus Representation Improved

Table 5 presents the results for gender differences in self-efficacy by women’s representation in STEM categories (i.e., underrepresented versus improved). Men had higher mean self-efficacy compared to women in both STEM domains. However, the gender difference was larger in the underrepresented than in the representation-improved fields, *t*(143) = 7.527, *p* < 0.00001, the 95% confidence intervals for the two STEM domains do not overlap. The mean effect size was small for STEM fields where representation-improved and medium for fields where underrepresentation persists. Both the underrepresented and representation-improved groups had significant variance in effect size that was not explained by artifacts (i.e., sampling and measurement error) suggesting the existence of other moderators. These potential moderators may be particularly relevant for the underrepresented STEM fields which had a larger variance of effects and a smaller proportion of variance explained by artifacts. Given the remaining unexplained variation, we explored whether the six specific STEM fields (i.e., biology, math, chemistry, engineering, physics, computer science) differed in their mean effect size. In other words, by focusing within each specific STEM field, can we account for the remaining variation in gender differences in self-efficacy?

##### Specific STEM Fields

**Figure 2:**
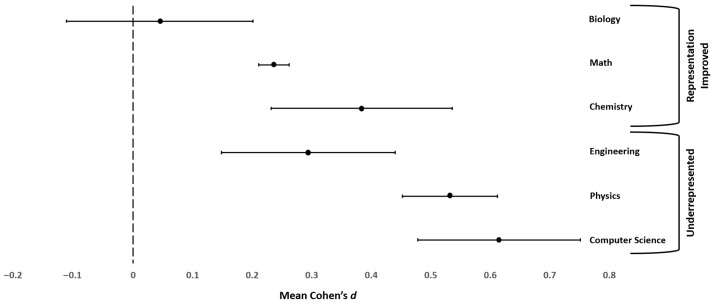
95% Confidence Intervals for Gender Differences in STEM Self-Efficacy by Specific STEM Field. *Note*. The 95% confidence interval is portrayed for each of the six STEM fields. The top three confidence intervals represent fields in which gender representation has improved; the bottom three confidence intervals represent STEM fields in which women continue to be underrepresented according to the Cheryan et al. (2017) classification. The filled circles represent the mean Cohen’s *d* for the STEM field. The lines extending from the mean to each end of the confidence interval (represented by a short vertical line) represent the margins of error. Negative values indicate females have greater mean self-efficacy than males in the STEM field and positive values indicate males have greater mean self-efficacy in the STEM field. The vertical dotted line indicates no gender difference in mean self-efficacy in the STEM field. Confidence intervals that do not overlap indicate that the mean effect size of the two groups differ at α = 0.01. When the overlap between the mean of one group and the error bar of the other group is less than 50%, the two groups differ at approximately α = 0.05, not corrected for multiple comparisons (Cumming & Finch, 2005).

Table 6 presents the meta-analysis results by specific STEM field. Figure 2 depicts the 95% confidence intervals for each of the six STEM fields.^7^ Although the Hunter–Schmidt method of meta-analysis does not recommend the use of significance testing to determine if effects differ across sub-groups (see Schmidt & Hunter, 2017), we conducted independent group t-tests using the meta-analysis results to provide more precise information about the statistical significance of the differences between subgroups. Table 7 provides the results for testing differences in gender difference in self-efficacy effect sizes by STEM field.

Biology had a mean *d* = 0.045 (95% CI [−0.112, 0.201]) indicating that the mean self-efficacy difference in biology for males and females did not differ from 0. All other STEM fields (see Table 6) were distinct from biology: the 95% confidence interval was centered around a positive value and did not overlap 0, indicating that males had higher self-efficacy than females. Recall that biology is the field where women have been equally represented since 1988.

Inspection of Figure 2 shows that small mean effect sizes were obtained indicating that males were higher than females in specific self-efficacy in math (*d* = 0.236) and engineering, *d* = 0.294. Chemistry showed a mean effect size between small and medium favoring men, *d* = 0.384. Finally, both physics (*d* = 0.532) and computer science (*d* = 0.615) showed a medium difference favoring men in specific self-efficacy. Math and chemistry are fields with improved representation of women over time, whereas engineering, physics, and computer science are fields where women continue to be underrepresented. The smaller than expected effect for engineering favoring men may reflect the heterogeneity of engineering specialties (e.g., electrical, mechanical versus biomedical engineering, environmental engineering) that vary in women’s representation. This possibility may be reflected in the relatively high variance of the d values for engineering and is explored further in the discussion. All six fields had remaining variation unaccounted for by artifacts. The percentage of the effect variance accounted for by artifacts alone ranged from 11.06% (engineering) to 45.92% (math; i.e., below the 75% cut-off). Although the effects vary by self-efficacy domain, there may be other important moderators. Given this remaining variation, we now consider the impact of participant educational stage as a potential explanation for the differences in effects.

##### Gender Differences in Self-Efficacy by Educational Stage

**Figure 3:**
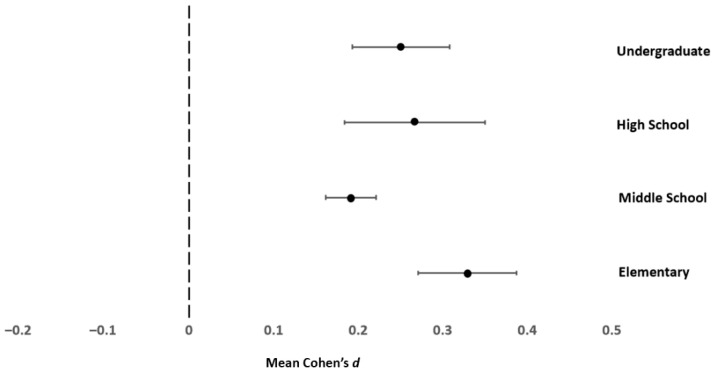
95% Confidence Intervals for Gender Differences in STEM Self-Efficacy by Educational Stage. *Note*. The 95% confidence interval is portrayed for each of the four educational stages.

Math was the only field with enough reported findings per educational stage to consider the potential moderation by educational stage (see Table 8). This outcome reflects the teaching of math classes at all grade levels, whereas biology, chemistry, engineering, and physics classes are generally only offered in high school or college level. As a more recent development, some computer science classes have been offered in high school and even earlier during the past several years. Table 9 provides the meta-analysis results by educational level and Table 10 provides the results of significance testing. At every educational level, the mean effect sizes showed a small or small to medium effect favoring men. Figure 3 depicts the 95% confidence intervals for math self-efficacy for the four educational levels. Inspection of Figure 3 suggests that the gender gap favoring men was smallest in middle school, and did not differ in elementary school, high school, or college.

Recall that math had the smallest initial variance in effect sizes (*σ*^2^ = 0.011) across the six STEM fields. After testing participant educational level as a moderator of gender differences in math self-efficacy the remaining variance unexplained by artifacts within middle school was no longer significant (i.e., over 75% of the variance in effects was explained by artifacts). This suggests that there are no other significant moderators in that educational stage. Additionally, in elementary school and undergraduate, nearly 60% of the remaining variance was explained by artifacts. In contrast, in high school, only 37% of the variance was attributable to artifacts and the variance was the highest among the educational levels. This finding suggests that high school may be a life stage that warrants additional exploration of factors that lead to varying gender differences in math self-efficacy.

### 3.3. Gender Differences by Year of Study

After checking for non-linearity and possible outliers, we computed Pearson correlations between the year of the study and the magnitude of the gender difference in self-efficacy by STEM field. The results showed that the magnitude of the gender difference decreased significantly over the years for chemistry (*r* = −0.712, *p* = 0.047, *n* = 8, range of years 2007 to 2019) and computer science (*r* = −0.574, *p* = 0.020, *n* = 16, range of years 1993 to 2021). Biology also showed a tendency to decrease that was between a medium and large effect size (*r* = −0.421, *p* = 0.173, *n* = 12, range of years 1999 to 2023), which did not attain statistical significance. Math (r = −0.220, *p* = 0.862, *n* = 67, range of years 1983 to 2023), physics (*r* = 0.104, *p* = 0.629, *n* = 24, range of years 2004 to 2022), and engineering (*r* = −0.070, *p* = 0.790, *n* = 18, range of years 1992 to 2029) showed no evidence of change over the years. These results indicate that the gender gap in self-efficacy has decreased over time in chemistry, computer science, and possibly biology, but remains largely unchanged over time in the other three STEM fields.

## 4. Discussion

### 4.1. Relationship Between Gender and STEM Self-Efficacy

#### 4.1.1. Domain-Specific Self-Efficacy

The present meta-analyses sought to build on previous findings and identify if gender differences in self-efficacy varied by women’s representation in STEM fields (i.e., were there larger differences in self-efficacy in STEM fields where the gender gap persists according to the Cheryan et al. (2017) classification?). To achieve this aim, the current research focused on studies that (1) measured field-specific self-efficacy (i.e., not general STEM or academic self-efficacy) and (2) measured the self-efficacy of participants who were currently students in the specific STEM field (e.g., not psychology students). These requirements minimized other sources of variability in domain-specific self-efficacy and allowed for a clearer understanding of the relationship between specific STEM fields and gender differences.

Through systematically synthesizing findings in the literature, the present meta-analytic work provided an empirical test of the hypotheses articulated in prior narrative reviews (e.g., Cheryan et al., 2017). This contribution moves the literature toward evidence-based assessment of gender-related patterns in STEM self-efficacy and where they are most pronounced. By identifying domains and educational stages in which gender gaps in self-efficacy are consistently present versus less pronounced, the findings offer guidance on where interventions may be most needed and how resources might be best allocated. The current findings serve as a bridge between prior narrative theory and future theoretical development. Meta-analytic synthesis, such as that conducted in the current research, is a necessary step for refining existing frameworks and providing a foundation upon which future theories can be built.

In line with the theoretical foundations of self-efficacy (Bandura, 1977, 1981; Cheryan et al., 2017), the meta-analysis findings suggested that gender differences in perceived competence in STEM were field specific. There were substantial differences in the magnitude of gender differences in self-efficacy by field. Our findings pointed to a relationship between women’s representation in a STEM field and the magnitude of the gender difference in self-efficacy in that field. Specifically, in computer science and physics—two of the STEM fields where the gender gap in participation remains—there was evidence of a substantially greater gender difference in self-efficacy as compared to the other fields (i.e., math, engineering). In contrast, in biology—a field where women currently earn over 60% of bachelor’s degrees—our findings suggested that self-efficacy did not differ by gender. This lack of mean gender difference in self-efficacy in biology may be due to the consistent representation of women in the field since 1988.

Although gender differences in computer science and physics self-efficacy were substantially greater than in the other fields, engineering—another field where women’s representation has traditionally been lacking—showed only a small gender difference. In fact, engineering had a substantially smaller average effect than the other two underrepresented STEM fields. Although the average effect in engineering was smaller, there was significant variation in that effect that was not accounted for by artifacts. This finding suggests that there may be potential moderators that influence gender differences in engineering self-efficacy.

One potential moderator may be the range of specialties in engineering (e.g., mechanical, electrical, biomedical, environmental). The relatively modest average effect size in engineering may reflect the aggregation of heterogeneous specialties with differing gender compositions and contextual dynamics. For example, some of these specialties have made greater improvements in women’s representation compared to others (e.g., degrees awarded to women in 2022: biomedical engineering (52.5%) and environmental engineering (48.9%) compared to electrical engineering (20.4%) and mechanical engineering (17.6%); American Society for Engineering Education, 2022). This heterogeneity may be theoretically meaningful, as it may highlight the importance of disciplinary context in shaping gendered experiences in engineering. Unfortunately, nearly all studies of gender differences in engineering self-efficacy considered engineering in general and not by engineering specialty. As such, there was not sufficient information to examine differences in the effect sizes across engineering specialties in the current meta-analysis. There is a critical need for future research efforts to focus on self-efficacy in specific engineering specialties to allow for comparisons across specialties where women remain underrepresented and specialties where representation has improved. This future research would allow for a clearer understanding of how structural, cultural, and instructional factors vary across engineering domains and how these differences shape gender differences in self-efficacy.

Although a major aim of this meta-analysis was to compare gender differences in self-efficacy across the six STEM fields, it is important to note that gender gaps in self-efficacy continue to exist in all but one of the STEM fields we considered (i.e., biology). These findings are in line with past meta-analyses that reported gender differences in self-efficacy in some specific STEM fields (e.g., computer science: Whitely, 1997; Huang, 2013; Parker et al., 2020; math: Hyde et al., 1990; Huang, 2013; Parker et al., 2020), but not biology (Parker et al., 2020). The current meta-analysis showed that gender differences in self-efficacy are largest in physics and computer science. Although this finding is important to our understanding of the persistent gender gap in STEM, it should not diminish the additional findings that there were also gender differences in self-efficacy in math, chemistry, and engineering. Biology, a field where women have been consistently represented for decades, was the only exception.

Small-to-medium effects, as found in all STEM fields other than biology, are meaningful at the population level, especially when they are observed consistently across large samples and STEM fields. Given that self-efficacy is strongly linked to many important outcomes (e.g., performance, interest, long-term persistence), it is still crucial for research to focus on bolstering women’s self-efficacy in nearly all STEM fields.^8^ Even modest gender differences in self-efficacy may have implications for important outcomes such as course selection, STEM field persistence, and career trajectories. This may be particularly true in educational and occupational pathways that involve repeated self-selection over time. This type of research may be particularly important for keeping women in the STEM pipelines beyond undergraduate degrees to graduate school and careers. On a positive note, our examination of the relationship of year of study and the magnitude of the gender gap indicated that the magnitude of the gender gap has decreased in chemistry and computer science.

Importantly, while self-efficacy is an established and influential predictor of STEM-related choices and outcomes, it is related to other psychological variables (e.g., belongingness, identity, and stereotypes) that were beyond the scope of the current research. The current analyses do not provide a comprehensive account of all mechanisms underlying gender gaps but synthesize and examine patterns in self-efficacy as a widely studied factor across STEM fields. Self-efficacy is often empirically and theoretically linked with other psychological variables. Gender-based variation in self-efficacy observed in this study may partially reflect the influence of these unmeasured variables. The relationship between self-efficacy and other psychological variables may be an important direction for future meta-analytic work that can disentangle the relative and interactive contributions of various psychological factors to the gender gap in STEM.

Additionally, the current meta-analysis focused on U.S.-based studies. This methodology was designed to reduce heterogeneity associated with cross-national differences such as educational systems, curriculum, and sociocultural norms related to gender and STEM. At the same time, gender gaps in STEM-related self-efficacy may differ across countries, which limits the direct generalizability of our findings outside of the U.S. The results should be interpreted as characterizing patterns within the U.S. rather than as universal features of gender differences in STEM. As such, cross-national meta-analytic work may be an important direction for future research. Studies might benefit from examining how structural, cultural, and policy-level factors may moderate gender gaps in STEM-related self-efficacy across countries.

#### 4.1.2. Life-Stage and Gender Differences in Self-Efficacy

Based on the stereotype threat and developmental perspectives, there was reason to consider the role of participant life stage in the magnitude of gender differences in STEM field self-efficacy. In the studies included in the current meta-analysis, math was the only field with enough studies of different educational stages to consider the role of participant educational stage in the magnitude of gender differences in STEM field self-efficacy. A prior meta-analysis considering math self-efficacy conducted over three decades ago found the gender gap in self-efficacy increased with increased schooling (Hyde et al., 1990). Additionally, other early research suggested that middle school was a critical point at which the gender difference in self-efficacy began to develop (e.g., Wigfield et al., 1991). This prior research may have stimulated the comparatively large number of effects considering math self-efficacy in middle school (*k* = 30) compared to elementary (*k* = 11), high school (*k* = 13), and undergraduate education (*k* = 8).

In the current research, there was a small to medium effect favoring men in self-efficacy at each educational level. The largest gender difference was in elementary school (*d* = 0.329, *σ*^2^ = 0.010), and the smallest was in middle school (*d* = 0.191, *σ*^2^ = 0.007). The gender difference in middle school math self-efficacy appeared to be smaller than in elementary school. Following middle school, the average gender difference was slightly larger than a small effect size for both high school (*d* = 0.267, *σ*^2^ = 0.023) and undergraduate students (*d* = 0.250, *σ*^2^ = 0.007). The overall findings of this analysis point to middle school as the period in which the smallest gender gap exists, perhaps indicating a crucial period to implement interventions to bolster women’s math self-efficacy, thereby preventing greater gender differences during later educational periods.

As noted in the results, math as a field had the smallest variance in effect sizes (*σ*^2^ = 0.011). After including participant educational level as a moderator, artifacts accounted for over 75% of the variance in effects in middle school and nearly 60% of the variance in elementary school and undergraduate education, indicating relatively little variation in the size of gender differences in self-efficacy. In contrast, in high school, only 37% of the variance was due to artifacts, suggesting that high school may be a stage that warrants additional research to explore factors that may moderate gender differences in math self-efficacy.

These educational-level-specific findings in math may be instructive for the design of future interventions and identifying areas of future research. Importantly, math as a field had the smallest initial variance, and it is a field where women have gained in representation. It is also the only STEM field that is part of the educational curriculum from kindergarten through college. The current study’s examination of educational stage in math serves as an illustrative and theoretically informative example for the other STEM fields. The findings serve to highlight the need for future research in other STEM fields. To the extent possible, future research should investigate gender differences in self-efficacy across educational levels in the other STEM fields. Outside of research on undergraduate students, very few studies measured field-specific self-efficacy for physics, engineering, computer science, biology, or chemistry. Of particular importance is to explore the precursors of self-efficacy and participation in fields where the gender gap is persistent in college undergraduates.

## 5. Conclusions

We conducted a quantitative review of the literature on gender differences in self-efficacy in six specific STEM fields that vary in women’s representation. To minimize sources of variability in domain specificity, we focused our review on studies that measured field-specific self-efficacy in participants currently involved in the STEM fields of interest. Using Hunter–Schmidt meta-analysis (Hunter & Schmidt, 1980; Schmidt & Hunter, 2017), we conducted a thorough review of the findings on the gender gap in these fields. We found that, with the exception of biology, small to medium differences between females and males in perceived self-efficacy persist. Fields in which women’s representation has improved, notably math and chemistry, showed only small effects of differences in perceived self-efficacy favoring men, whereas fields in which women’s underrepresentation persists, notably physics and computer science, showed medium effects favoring men. Engineering, a STEM field that has been categorized as having an underrepresentation of women, showed only a small effect size favoring men, possibly because of the large differences in the proportion of women enrolled in the different subfields of engineering. The effect size of the difference favoring men in perceived self-efficacy has decreased over time in chemistry and computer science, but not in the other fields.

## Figures and Tables

**Table 1:** Criteria for Inclusion in Meta-Analysis.

Criteria	Description
Quantitative Measures and Statistical Information	Studies must include quantitative measures of gender composition of sample and self-efficacy in STEM. Each included study must have a quantitative result reported for the relationship between gender and the self-efficacy variable. For example, the study could report mean self-efficacy values for males and females, a correlation between gender and the self-efficacy variable, a regression coefficient between gender and self-efficacy. Measures of self-efficacy were defined as measures that assessed an individual’s judgments about their skills and abilities to succeed.
STEM Field Specific	Studies must focus on one or more of the STEM fields where women are currently underrepresented (i.e., computer science, engineering, physics) and/or STEM fields where women’s representation has improved (i.e., math, chemistry, biology). Study sample must be currently involved in the specific STEM field (e.g., enrolled in a Chemistry course; Chemistry majors; Chemistry professionals).
Participants	To allow for comparisons in self-efficacy, samples must include both male and female participants.
Date of Publication	No restrictions for publication date.
Publication Type	Publications can include peer-reviewed articles and peer-reviewed conference papers.
Language	Full-text must be available in English.
Location	To limit differences due to structure of the education system and national culture, samples must be located within the United States.
New and Original Data	Systematic reviews, meta-analyses, and literature reviews that do not present new data findings were excluded.

**Table 2:** Reported Effects by STEM Field.

	Articles	Computer Science	Engineering	Physics	Biology	Chemistry	Math	Total Effects
SE Gender Comparison	109	16	17	24	12	8	67	144

*Note*. SE = Self-Efficacy. One reported effect was for two underrepresented STEM fields combined (rather than each separately). This result was excluded from the meta-analyses that considered individual STEM fields as the moderator. Total effects for self-efficacy gender comparison = 145, after exclusion of combined effect, *n* = 144. Five articles contained multiple effects for the same construct. Those effects were averaged to create one effect (see Cooper, 2017, pp. 142–149 for a discussion). Twelve articles contained two or more longitudinal effects. Nine articles included more than one study or multiple effects for different constructs (e.g., chemistry self-efficacy and biology self-efficacy). Each of those effects was included separately so that total effects exceed that number of articles.

**Table 3:** Significance of Reported Self-Efficacy Gender Difference Findings by STEM Domain.

		Male Greater than Female	Female Greater than Male	No Significant Difference	Total Number of Effects
Representation Improved	Biology	4 (33.3%)	1 (8.3%)	7 (58.3%)	12
	Chemistry	5 (62.5%)	0 (0%)	3 (37.5%)	8
	Math	43 (64.2%)	1 (1.5%)	23 (34.3%)	67
	Totals	52 (59.8%)	2 (2.3%)	33 (37.9%)	87
Underrepresented	Computer Science	12 (75.0%)	0 (0%)	4 (25.0%)	16
	Physics	23 (95.8%)	0 (0%)	1 (4.2%)	24
	Engineering	8 (47.1%)	1 (5.9%)	8 (47.1%)	17
	Totals	43 (75.4%)	1 (1.8%)	13 (22.8%)	57

*Note*. Significant differences based on the *p*-values reported in the articles.

**Table 4:** Meta-Analysis Results for Gender Differences in Self-Efficacy for All STEM Fields Combined.

All STEM Fields Combined	
# of Effects (*k*)	145
Mean d-Value	0.308
95% Confidence Interval	0.273–0.342
80% Credibility Interval	0.050–0.566
Variance of d-Values	0.045
Variance due to Artifacts	0.005
Percent Variance due to Artifacts	10.1%
Bare Bones Mean d-Value	0.277
Bare Bones Variance of d-Values	0.031

*Note*. Artifacts = sampling error and measurement error. *d* represents Cohen’s (1988) *d*. A positive Cohen’s *d* indicates that the mean for men was greater than the mean for women. Bare Bones Mean d-Value = mean d-value correcting only for sampling error. Bare Bones Variance of d-Values = variance of d-values after correcting for sampling error.

**Table 5:** Meta-Analysis Results for Gender Differences in STEM Self-Efficacy by Women’s Representation.

	Underrepresented	Representation Improved
# of Effects (*k*)	58	87
Mean d-Value	0.521	0.234
95% Confidence Interval	0.451–0.590	0.207–0.262
80% Credibility Interval	0.201–0.840	0.101–0.368
Variance of d-Values	0.073	0.017
Variance due to Artifacts	0.010	0.006
Percent Variance due to Artifacts	14.32%	36.62%
Bare Bones Mean d-Value	0.469	0.210
Bare Bones Variance of d-Values	0.041	0.009

*Note*. *k* = number of effects in the meta-analysis. Artifacts = sampling error and measurement error. *d* = Cohen’s (1988) *d*. A positive Cohen’s *d* indicates that the mean for men was greater than the mean for women. Following Cheryan et al. (2017), underrepresented STEM fields are engineering, physics, and computer science. Representation-improved STEM fields include biology, math, and chemistry. Bare Bones Mean d-Value = mean d-value correcting only for sampling error. Bare Bones Variance of d-Values = variance of d-values after correcting for sampling error.

**Table 6:** Meta-Analysis Results for Gender Differences in STEM Self-Efficacy by Specific STEM Field.

	Representation Improved			Underrepresented		
	Biology	Math	Chemistry	Engineering	Physics	Computer Science
# of Effects (*k*)	12	67	8	17	24	16
Mean d-Value	0.045	0.236	0.384	0.294	0.532	0.615
95% Confidence Interval	−0.112–0.201	0.211–0.262	0.232–0.536	0.148–0.440	0.452–0.612	0.478–0.751
80% Credibility Interval	−0.250–0.339	0.136–0.336	0.156–0.612	−0.060–0.648	0.308–0.756	0.278–0.951
Variance of d-Values	0.077	0.011	0.048	0.094	0.040	0.078
Variance due to Artifacts	0.024	0.005	0.017	0.018	0.009	0.009
% Variance due to Artifacts	30.80%	45.92%	34.29%	19.01%	22.55%	11.06%
Bare Bones Mean d-Value	0.046	0.211	0.355	0.268	0.491	0.527
Bare Bones Variance	0.046	0.005	0.022	0.060	0.020	0.038

*Note*. *k* = number of effects in the meta-analysis. Artifacts = sampling error and measurement error. *d* = Cohen’s (1988) *d*; A positive Cohen’s *d* indicates that the mean for men was greater than the mean for women; Bare Bones Mean d-Value = mean d-value correcting only for sampling error; Bare Bones Variance = variance of d-values after correcting for sampling error.

**Table 7:** Significant Differences in Gender Differences in Self-Efficacy Effect Sizes by STEM Field.

	Biology	Chemistry	Math	Physics	Engineering
Biology Mean d-Value: 0.045 95CI: −0.112–0.201					
Chemistry Mean d-Value: 0.384 95CI: 0.232–0.536	*t*(18) = 2.901, *p* = 0.010				
Math Mean d-Value: 0.236 95CI: 0.211–0.262	***t*(77) = 4.265,** ***p* < 0.0001**	***t*(73) = 3.278,** ***p* = 0.002**			
Physics Mean d-Value: 0.532 95CI: 0.452–0.612	***t*(34) = 6.047,** ***p* < 0.0001**	*t*(30) = 1.772, *p* = 0.087	***t*(89) = 9.145,** ***p* < 0.0001**		
Engineering Mean d-Value: 0.294 95CI: 0.148–0.440	*t*(27) = 2.238, *p* = 0.034	*t*(23) = 0.741, *p* = 0.466	*t*(82) = 1.294, *p* = 0.200	*t*(39)= 3.009, *p* = 0.005	
Computer Science Mean d-Value: 0.615 95CI: 0.478–0.751	***t*(26) = 5.366,** ***p* < 0.0001**	*t*(22) = 2.041, *p* = 0.054	***t*(81) = 8.904,** ***p* < 0.0001**	*t*(38) = 1.100, *p* = 0.280	*t*(31) = 3.137, *p* = 0.004

*Note*. *p*-values are based on separate *t*-tests for each effect. Bolded *p*-values indicate a significant difference in Cohen’s d between STEM fields following the Bonferoni adjustment of α for multiple tests. Adjusted Bonferoni α = 0.05/15 = 0.003.

**Table 8:** Reported Effects for Gender Differences in Self-Efficacy by Educational Level.

	Elementary	Middle School	High School	Undergraduate	Grad School	Career
Computer Science	1	4	1	6	1	-
Engineering	1	-	-	13	-	1
Physics	-	-	-	22	-	-
Biology	-	-	2	7	1	-
Chemistry	-	-	-	7	-	-
Math	11	30	13	8	-	-

**Table 9:** Meta-Analysis Results for Gender Differences in Math Self-Efficacy by Life Stage.

	Life Stages		
	Elementary	Middle School	High School
# of Effects (*k*)	11	30	13
Mean d-Value	0.329	0.191	0.267
95% Confidence Interval	0.271–0.387	0.162–0.221	0.184–0.350
80% Credibility Interval	0.250–0.408	0.142–0.241	0.111–0.423
Variance of d-Values	0.010	0.007	0.023
Variance due to Artifacts	0.006	0.005	0.009
% Variance due to Artifacts	59.68%	77.72%	36.79%
Bare Bones Mean d-Value	0.300	0.170	0.244
Bare Bones Variance	0.003	0.002	0.013

*Note*. *k* = number of effects in the meta-analysis. Artifacts = sampling error and measurement error. *d* = Cohen’s (1988) *d*. Negative values indicate females have greater mean self-efficacy than males and positive values indicate males have greater mean self-efficacy. Bare Bones Mean d-Value = mean d-value correcting only for sampling error. Bare Bones Variance = variance of d-values after correcting.

**Table 10:** Significant Differences in Gender Differences in Math Self-Efficacy Effect Sizes by Life Stage.

	Elementary	Middle School	High School	Undergraduate
Elementary Mean d-Value: 0329 95CI: 0.271–0.387				
Middle School Mean d-Value: 0.191 95CI: 0.162–0.221	***t*(39) = 4.430,** ***p* < 0.0001**			
High School Mean d-Value: 0.267 95CI: 0.184–0.350	*t*(22) = 1.156, *p* = 0.260	*t*(41) = 2.111, *p* = 0.041		
Undergraduate Mean d-Value: 0.250 95CI: 0.193–0.308	*t*(17) = 1.814, *p* = 0.087	*t*(36) = 1.765, *p* = 0.086	*t*(19) = 0.289, *p* = 0.776	

*Note*. *p*-values are based on separate *t*-tests for each effect. Bolded *p*-values indicate a significant difference in Cohen’s d between STEM fields following the Bonferoni adjustment of α for multiple tests. Adjusted Bonferoni α = 0.05/6 = 0.008.

## Data Availability

The data are available to download at https://osf.io/jt3a4/?view_only=3109a214794d406b8a3e2ef0837d8a43.

## Notes

1 These databases were recommended by research librarians at the researcher’s university in order to access articles related to psychological constructs (e.g., self-efficacy), education, and specific STEM fields. The searches were completed in May 2023.

2 Online Resource S1 is available at https://osf.io/jt3a4/?view_only=3109a214794d406b8a3e2ef0837d8a43. Citations for all articles included in the meta-analysis are available in Supplementary F in Online Resource S1.

3 One hundred and five abstracts were identified by coding team members but were not identified by the first author. The first author reviewed the full-text of all identified abstracts (Abstracts Identified by First Author, *n* = 707; Unique Abstracts Identified by Coding Team, *n* = 105; Total Full-Text Articles Reviewed Based on Identified Abstracts, *N* = 812; see the flow chart in Figure 1).

4 See Schmidt and Hunter (2017) for details regarding how the corrected effect sizes, confidence intervals, and credibility intervals are calculated.

5 In articles in which the actual year the study was conducted was reported, we used that value for the date of the study. In articles in which the year of data collection was not reported, we used publication date as a proxy for year of data collection.

6 The Hunter–Schmidt method of meta-analysis does not recommend the use of significance testing to determine if effects differ across sub-groups (see Schmidt & Hunter, 2017). For purposes of quantifying the differences in effect sizes between groups, after computing the meta-analysis statistics by sub-groups, we calculated 95% confidence intervals to provide a description of the effect size of each of the groups and to provide information for approximate statistical inference regarding differences between the groups (Cumming & Finch, 2005; Cumming, 2012). Additionally, we conducted independent group t-tests using the meta-analysis results to provide a clearer understanding of the significance of the differences between subgroups.

7 Forest plots depicting the meta-analyses findings are presented in Online Resource S1. The plots present the effects by sample size in descending order allowing for identification of potential outliers and publication bias (i.e., effect sizes that are systematically larger as sample sizes decrease; Schmidt & Hunter, 2017). In random effects models for meta-analysis, it can be problematic to distinguish between outliers and true, extreme effects especially if the sample size for the potential outlier effect is small (Schmidt & Hunter, 2017; Baker & Jackson, 2008). Erroneously eliminating true, deviant effects can lead to artificially small estimated variance in the meta-analysis effect size (for a detailed discussion of this issue see Schmidt & Hunter, 2017). As such, the current meta-analysis created forest plots to visualize the data points and identify evidence of very extreme data points that could be due to error (Schmidt & Hunter, 2017). Inspection of the plots did not reveal extreme data points that were clear outliers. As such, all effects were included in the meta-analyses.

8 See Supplementary E in Online Resource S1 for additional exploratory meta-analyses on the relationship between self-efficacy and interest and persistence in STEM fields by women’s representation.

## Supplementary Materials

The following supporting information can be downloaded at: https://osf.io/jt3a4/?view_only=3109a214794d406b8a3e2ef0837d8a43.
